# Fear of COVID-19 and PTSD symptoms in adolescents: the mediating role of meaning in life and the moderating role of gender

**DOI:** 10.3389/fpsyg.2026.1912062

**Published:** 2026-10-23

**Authors:** Wen Lv, Yuguang Wang, Zeling Long, Shienyu Fei, Juan Song, Wei Chen

**Affiliations:** 1 School of Psychology, Guizhou Normal University, Guiyang, China; 2 Key Laboratory of Brain Function and Brain Disease Prevention and Treatment of Guizhou Province, Guiyang, China; 3 School of Public Administration, Sichuan University, Chengdu, China; 4 Shizhen College of Guizhou University of Traditional Chinese Medicine, Guiyang, China

**Keywords:** adolescents, fear of COVID-19, gender, meaning in life (MiL), post-traumatic stress disorder (PTSD)

## Abstract

**Background:**

The COVID-19 pandemic and related restrictions were associated with substantial mental health burdens among adolescents, including PTSD symptoms. Meaning in life may explain the association between fear of COVID-19 and PTSD symptoms, and this association may differ by gender.

**Objective:**

This study examined the association between fear of COVID-19 and PTSD symptoms among Chinese adolescents, tested the distinct indirect effects of Presence of Meaning and Search for Meaning, and evaluated whether gender moderated the second-stage paths from both meaning dimensions to PTSD.

**Methods:**

A cross-sectional study was conducted with 1,291 middle school students recruited through convenience sampling. Participants completed the FCV-19S, PCL-C, MLQ, and a demographic survey. The sample included 656 females (50.8%) and 635 males (49.2%), aged 11–18 years (M = 14.13, SD = 1.35). Analyses were conducted using IBM SPSS 27.0 and PROCESS macro version 4.1; age was included as a covariate in every mediation and moderated mediation equation.

**Results:**

Fear of COVID-19 was positively correlated with PTSD symptoms (*r* = 0.31, *p* < 0.001). After controlling for age, the indirect effect was significant through Presence of Meaning (*B* = 0.059, 95% CI [0.031, 0.089]) but not Search for Meaning (*B* = 0.005, 95% CI [−0.001, 0.015]); this pattern held in the parallel model (Presence: *B* = 0.060, 95% CI [0.032, 0.092]; Search: *B* = −0.002, 95% CI [−0.009, 0.004]). Gender moderated the second-stage Presence–PTSD path (*B* = −0.159, *p* = 0.044; stronger among females) and the Search–PTSD path (*B* = −0.231, *p* = 0.016; negative among females but not males). For Presence of Meaning, conditional indirect effects were significant for both males and females, whereas the index of moderated mediation was not significant (index = 0.012, 95% CI [−0.002, 0.034]); for Search for Meaning, conditional indirect effects were not significant.

**Conclusion:**

In age-adjusted models, Presence of Meaning, but not Search for Meaning, showed a significant indirect association. Gender moderated the second-stage paths from both dimensions to PTSD, but the overall indirect effects did not differ by gender. These findings support distinguishing having meaning from seeking meaning; longitudinal research is needed to test causal or intervention implications.

## Introduction

The COVID-19 pandemic and related restrictions were associated with substantial mental health burdens across populations (Casagrande et al., 2020; Houben-Wilke et al., 2022). Adolescents may be especially vulnerable during public health crises because of developmental changes and disruptions to social and academic routines (Sayed et al., 2021; Sultana et al., 2021). PTSD symptoms were one concern, although generalized pandemic threat does not necessarily satisfy DSM-5 Criterion A for PTSD (APA, 2013). Fear of infection, severe illness, or loss of loved ones was a plausible correlate of distress, and prior disaster and pandemic studies have linked fear or perceived threat with PTSD symptoms (Cao et al., 2019; Mertens et al., 2020). The present study therefore examined how fear of COVID-19 was associated with PTSD symptoms among adolescents and whether meaning in life helped account for that association.

Emotional processing theory provides a rationale for expecting an association between fear and PTSD symptoms by proposing that pathological fear structures are central to PTSD (Foa and Kozak, 1986). In a pandemic context, repeated threat cues and persistent virus-related fear may be associated with maladaptive threat representations (Mertens et al., 2020). Neurobiological research has also linked PTSD with heightened threat responding and altered prefrontal regulation (Kemp et al., 2007). Because the present data are cross-sectional, this literature was used to motivate a test of association rather than to assume that fear caused PTSD symptoms. Accordingly, H1 predicted a positive association between fear of COVID-19 and PTSD symptoms.

The association between fear and PTSD symptoms may also involve psychological resources. Terror management theory describes how existential threat can challenge anxiety-buffering beliefs (Pyszczynski et al., 2015), whereas meaning-making theory emphasizes efforts to integrate stressful experiences within a coherent system of meaning (Park, 2010). A stable sense of meaning has been associated with resilience and fewer negative emotional or post-traumatic symptoms (Arslan et al., 2022; Fischer et al., 2023; Zhong et al., 2019). The MLQ distinguishes Presence of Meaning, an existing sense of purpose and coherence, from Search for Meaning, the active pursuit of meaning (Steger et al., 2006). We therefore tested these dimensions separately as potential mediators rather than treating meaning in life as a single undifferentiated construct.

Associations among trauma responses, meaning, and adjustment may vary across individuals. Gender differences have been reported in PTSD-related responses and in adolescent peer-relationship processes (Kimerling et al., 2018; Rahman et al., 2021; Rose and Rudolph, 2006). These findings do not establish that girls and boys derive meaning from different sources, but they provide a rationale for testing whether the association between meaning in life and PTSD symptoms differs by gender. We therefore treated gender moderation as an empirical question rather than assuming a gender-specific psychological mechanism.

Consequently, the study tested whether gender moderated the second-stage association between each meaning dimension and PTSD symptoms. Conditional indirect effects were evaluated separately from the interaction term so that evidence for moderation of one path would not be interpreted automatically as evidence for moderated mediation.

### The present study

Previous research has linked pandemic-related fear, meaning in life, and PTSD symptoms, but the distinct roles of Presence of Meaning and Search for Meaning remain insufficiently understood in adolescents. The present study examined their simple and unique parallel indirect effects and then evaluated gender as a moderator of the second-stage meaning-PTSD paths. The analyses were framed as tests of cross-sectional associations; they were not intended to establish temporal or causal mechanisms.

By separating the two MLQ dimensions and reporting both the interaction and the index of moderated mediation, this study may clarify which proposed associations are supported and which remain uncertain. Any implications for intervention are therefore considered provisional and require confirmation in longitudinal or experimental research.

Based on the theoretical rationale outlined above, we proposed the following hypotheses, which are summarized in our conceptual model:

H1: Fear of COVID-19 would be positively associated with PTSD symptoms.

H2a: Presence of Meaning would mediate the association between fear of COVID-19 and PTSD symptoms.

H2b: Search for Meaning would mediate the association between fear of COVID-19 and PTSD symptoms.

H3a: Gender would moderate the second-stage Presence of Meaning–PTSD association.

H3b: Gender would moderate the second-stage Search for Meaning–PTSD association.

## Methods

### Participants and procedure

#### Participants

A cross-sectional study was conducted using convenience sampling. Four middle schools were selected on the basis of accessibility and administrative approval in Guizhou, Henan, and Hubei provinces, China. A total of 1,400 students were approached. No students were excluded under exclusion criterion 1. Nineteen were excluded because they reported significant pre-pandemic trauma (criterion 2), 49 because more than 10% of data on the primary scales were missing (criterion 3), and 41 because questionnaires were incomplete. The final sample comprised 1,291 students (response rate = 92.2%). Gender was assessed using a single self-report item with the response options “male” and “female.” The sample included 656 females (50.8%) and 635 males (49.2%), aged 11–18 years (M = 14.13, SD = 1.35).

#### Inclusion and exclusion criteria

To ensure sample homogeneity and enhance the internal validity of the findings, specific inclusion and exclusion criteria were applied.

The inclusion criteria were as follows: (1) aged above 10 years; (2) capable of understanding and completing the self-report questionnaires independently; (3) provision of written informed consent from a legal guardian, alongside verbal assent from the participant themselves.

The exclusion criteria were: (1) *a prior* diagnosis of a severe mental disorder (e.g., schizophrenia, bipolar disorder) by a professional medical institution, to minimize confounding effects of pre-existing conditions; (2) a self-reported history of significant traumatic experiences (e.g., natural disasters, abuse) occurring before the COVID-19 pandemic, to increase the likelihood that the assessed PTSD symptoms were related to the pandemic context; (3) submission of a questionnaire with more than 10% of data missing on any of the primary scales.

#### Procedure

This study was approved by the Ethics Committee of the School of Psychology, Guizhou Normal University. Data were collected between November and December 2022. Written informed consent was obtained from participants’ legal guardians, verbal assent was obtained from participants, and administrative approval was secured from the participating schools. Participants were informed that their data would be used for scientific research and handled in accordance with standard research confidentiality practices. During organized sessions, participants completed standardized self-report measures. For participants who screened positive for probable PTSD (PCL-C score ≥ 50), the research team adopted a minimal-disclosure approach. School counselors were informed in general terms that some students might benefit from available psychological support. Legal guardians were provided with information about school-based support resources through routine school communication channels. No specific screening scores or clinical details were disclosed, and the decision to seek further support was left entirely to the guardians.

### Measures

#### Fear of COVID-19 scale

Fear of COVID-19 was assessed using the FCV-19S (Ahorsu et al., 2022), a seven-item self-report scale. Each item is rated from 1 (“strongly disagree”) to 5 (“strongly agree”). An example item is “I cannot sleep because I’m worrying about getting COVID-19.” Total scores range from 7 to 35, with higher scores indicating greater fear of COVID-19. Cronbach’s alpha was 0.90 in the present sample.

#### PTSD checklist–civilian version

PTSD symptoms were assessed using the 17-item PCL-C (Andrykowski et al., 1998). Participants were instructed to answer with reference to COVID-19-related experiences, although the PCL-C does not anchor responses to a specific DSM-5 Criterion A event. Items are rated on a five-point scale, producing total scores from 17 to 85; higher scores indicate greater symptom severity. A score of 50 or higher was used only as a screening threshold for probable PTSD and not as a clinical diagnosis. The Chinese PCL-C has demonstrated reliability and validity (Yang et al., 2007). Cronbach’s alpha was 0.93 in the present sample.

#### Meaning in life questionnaire

The MLQ was developed by Steger et al. (2006); the present study used the Chinese revision by Chen et al. (2015). The corresponding author, Wei Chen, is a co-author of that revision. The 10-item scale contains two five-item subscales: Presence of Meaning (PM) and Search for Meaning (SM). Items are rated from 1 (“completely non-conforming”) to 7 (“completely conforming”). In the Chinese revision used here, Presence of Meaning comprised items 2, 4, 7, 9, and 10, with item 2 reverse-scored. Search for Meaning comprised items 1, 3, 5, 6, and 8. Higher PM scores indicate a clearer existing sense of meaning and purpose, whereas higher SM scores indicate more active pursuit of meaning and do not by themselves indicate a lack of meaning. The Cronbach’s alpha for the total scale was 0.86. Cronbach’s alpha was 0.90 for PM and 0.86 for SM in the present sample.

All scale scores were computed according to the original scoring instructions and the Chinese revision of the MLQ. Item-to-dimension assignments, reverse-scoring, and total-score computation were verified before the final analyses. Data cleaning, missing-data imputation, and all scoring procedures are documented in Supplementary Materials 1, 3, 4, and 5.

### Data analysis

IBM SPSS version 27.0 and PROCESS macro version 4.1 (Hayes, 2022) were used. Item-level missingness on the self-report measures was less than 2%. After excluding cases with more than 10% missing data on the primary scales, the remaining missing values were handled using stochastic regression single imputation (Schafer and Graham, 2002). This procedure created one completed dataset, and all descriptive, PROCESS, and MG-CFA analyses reported below were conducted on that same dataset. Because single imputation does not incorporate imputation uncertainty, this choice is also acknowledged as a limitation.

To examine whether the MLQ measured the same constructs across gender groups, measurement invariance was tested using the lavaan package in R (Rosseel, 2012). Configural, metric, and scalar models were estimated sequentially within a consistent multi-group framework. Because tests indicated non-normality (Kolmogorov–Smirnov and Shapiro–Wilk tests for all variables, *p* < 0.001), robust maximum likelihood (MLR) estimation was used to obtain robust standard errors and fit indices. Complete fit statistics are reported in the Results section. Full lavaan syntax and output are provided in Supplementary Material 6.

Descriptive statistics and bivariate correlations were computed first. Two simple mediation models and one parallel mediation model were estimated using PROCESS Model 4. The planned conditional process analysis used PROCESS Model 14 (Hayes, 2015, 2022), with gender moderating the second-stage path. Age was included as a covariate in every mediator and outcome equation. In Model 14, gender and the mediator × gender product term entered the PTSD outcome equation but not the mediator equation; consequently, the fear-to-mediator a path was estimated across the full sample. Gender was dummy coded (male = 0, female = 1). Indirect effects and the index of moderated mediation were evaluated using percentile bootstrap confidence intervals based on 5,000 resamples (Shrout and Bolger, 2002); intervals excluding zero were interpreted as statistically significant. Significant interactions were probed using simple-slope analyses (Aiken et al., 1991).

## Results

### Test for common method bias

As the data for multiple variables in this study were provided by the same participants through self-report questionnaires, there was a risk of common method bias. The Harman single-factor test was conducted as a preliminary statistical check (i.e., principal component factor analysis of all items without rotation) (Zhou and Long, 2004). The results showed that a total of six factors had eigenvalues greater than 1 after non-rotated analysis, and the largest factor explained 27.83% of the variance, below the conventional 40% threshold. This provides basic reassurance that common method bias is not a pervasive concern in our data. However, we acknowledge the limited sensitivity of this test and address this in the Limitations section.

### Preliminary analyses

Table 1 presents the means, standard deviations, and correlations among the study variables. As shown in Table 1, female adolescents reported slightly higher PTSD symptoms (M = 28.07, SD = 11.43) and fear of COVID-19 (M = 15.72, SD = 5.96) compared to males (PTSD: M = 26.68, SD = 11.13; fear: M = 14.77, SD = 6.37). In contrast, males reported slightly higher Presence of Meaning (M = 24.19, SD = 7.37) and Search for Meaning (M = 26.56, SD = 6.50) than females (Presence: M = 22.91, SD = 7.27; Search: M = 25.72, SD = 5.97). Fear of COVID-19 was positively correlated with PTSD (*r* = 0.31, *p* < 0.001) and gender (*r* = 0.08, *p* = 0.006) and negatively correlated with Presence of Meaning (r = −0.13, *p* < 0.001). Its correlation with Search for Meaning was not significant (*r* = −0.05, *p* = 0.051). Presence of Meaning and Search for Meaning were both negatively correlated with PTSD (*r* = −0.28, *p* < 0.001, and *r* = −0.07, *p* = 0.010, respectively) and with gender (*r* = −0.09, *p* = 0.002, and *r* = −0.07, *p* = 0.015, respectively). Presence of Meaning was positively correlated with Search for Meaning (*r* = 0.29, *p* < 0.001). Age was negatively correlated with PTSD (*r* = −0.08, *p* = 0.005), positively correlated with Search for Meaning (*r* = 0.06, *p* = 0.027), and was not significantly correlated with fear of COVID-19 (*r* = −0.05, *p* = 0.052), Presence of Meaning (*r* = −0.02, *p* = 0.501), or gender (*r* = −0.04, *p* = 0.144). These patterns provided preliminary support for H1 and warranted age-adjusted tests of the proposed mediation models.

**TABLE 1:** Descriptive statistics and correlations between variables (*N* = 1,291).

Variables	*M ± SD*	Male	Female	1	2	3	4	5	6
1. Gender				1					
2. Age	14.13 ± 1.35	14.18 ± 1.39	14.07 ± 1.31	−0.04	1	1	1	1	1
3. PTSD	27.38 ± 11.30	26.68 ± 11.13	28.07 ± 11.43	0.06*	−0.08**				
4. Fear of COVID-19	15.25 ± 6.18	14.77 ± 6.37	15.72 ± 5.96	0.08**	−0.05	0.31***			
5. Presence of Meaning	23.54 ± 7.34	24.19 ± 7.37	22.91 ± 7.27	−0.09**	−0.02	−0.28***	−0.13***		
6. Search for Meaning	26.13 ± 6.25	26.56 ± 6.50	25.72 ± 5.97	−0.07*	0.06*	−0.07*	−0.05	0.29***	

Gender was coded male = 0 and female = 1; correlations involving gender are point-biserial. 1 = Gender; 2 = Age; 3 = PTSD; 4 = Fear of COVID-19; 5 = Presence of Meaning; 6 = Search for Meaning.

*p < 0.05.

**p < 0.01.

***p < 0.001.

### Measurement invariance of the MLQ

Measurement invariance of the MLQ across gender was tested within a consistent multi-group framework using the lavaan package in R (Rosseel, 2012). A series of increasingly restrictive models–configural, metric, and scalar–were estimated sequentially on the full sample (N = 1, 291; 635 males, 656 females). Robust maximum likelihood (MLR) estimation was used. Model fit was evaluated using the comparative fit index (CFI), Tucker–Lewis index (TLI), root mean square error of approximation (RMSEA), and standardized root mean square residual (SRMR). Changes in CFI (ΔCFI ≤ 0.010) and RMSEA (ΔRMSEA ≤ 0.015) were used to compare nested models (Chen, 2007).

The configural model, which allowed all parameters to vary freely across groups, showed acceptable fit: χ^2^(68) = 534.716, CFI = 0.936, TLI = 0.916, RMSEA = 0.103, SRM*R* = 0.055. This indicates that the two-factor structure of the MLQ was supported in both male and female adolescents.

When factor loadings were constrained to be equal across groups (metric invariance), the model fit remained stable: χ^2^(76) = 546.026, CFI = 0.936, TLI = 0.924, RMSEA = 0.098, SRMR = 0.058; ΔCFI = −0.001, ΔRMSEA = −0.005. Both changes were within the recommended cutoffs, supporting metric invariance.

When item intercepts were further constrained to be equal across groups (scalar invariance), the model fit did not worsen: χ^2^(84) = 559.454, CFI = 0.935, TLI = 0.930, RMSEA = 0.094, SRMR = 0.058; ΔCFI = −0.001, ΔRMSEA = −0.004. These changes were also within the recommended cutoffs, supporting scalar invariance.

Together, these results support scalar invariance of the two-factor MLQ across gender, permitting comparison of the observed subscale means and of the regression slopes across genders. The configural model fit was acceptable but not optimal (RMSEA = 0.103), a point considered in the Limitations section. Full lavaan syntax and output are provided in the Supplementary Material.

### Testing for mediation effects

PROCESS Model 4 was used to test the two simple mediation models and the parallel mediation model, with age included as a covariate in all equations. Unstandardized coefficients are reported. The simple mediation results are presented in Tables 2, 3 and 4.

**TABLE 2:** Simple mediation models: presence of meaning.

Variable	PTSD			Presence of meaning			PTSD		
	*B*	*t*	*95% CI*	*B*	*t*	*95% CI*	*B*	*t*	*95% CI*
Presence of meaning model									
Fear of COVID-19	0.557	11.504***	[0.462,0.652]	−0.157	−4.788***	[−0.222, −0.093]	0.499	10.540***	[0.406, 0.591]
Presence of Meaning							−0.373	−9.390***	[−0.452, 0.295]
Age	−0.514	−2.312*	[−0.949, −0.078]	−0.141	−0.937	[−0.437, 0.154]	−0.566	−2.633**	[−0.988, −0.144]
*R* ^2^	0.099			0.018			0.156		
*F*	70.482***			11.694***			79.559***		

B = unstandardized regression coefficient; CI = 95% confidence interval. Age was included as a covariate in every equation.

*p < 0.05.

**p < 0.01.

***p < 0.001.

**TABLE 3:** Simple mediation models: search for meaning.

Variable	PTSD			Search for meaning			PTSD		
	*B*	*t*	*95% CI*	*B*	*t*	*95% CI*	*B*	*t*	*95% CI*
Search for meaning model									
Fear of COVID-19	0.557	11.504***	[0.462, 0.652]	−0.052	−1.840	[−0.107, 0.003]	0.553	11.401***	[0.457, 0.648]
Search for meaning							−0.093	−1.939	[−0.187, 0.001]
Age	−0.514	−2.312*	[−0.949, −0.078]	0.272	2.108*	[0.019, 0.525]	−0.488	−2.196*	[−0.924, −0.052]
*R* ^2^	0.099			0.006			0.101		
*F*	70.482***			4.137*			48.342***		

B = unstandardized regression coefficient; CI = 95% confidence interval. Age was included as a covariate in every equation.

*p < 0.05.

**p < 0.01.

***p < 0.001.

**TABLE 4:** Indirect effects in simple mediation models.

Variable	Effect (*B*)	BootSE	BootLLCI	BootULCI
Presence of meaning	0.059	0.015	0.031	0.089
Search for meaning	0.005	0.004	−0.001	0.015

Effect (B) = unstandardized indirect effect; BootSE = bootstrap standard error; BootLLCI and BootULCI are the lower and upper limits of the 95% percentile bootstrap confidence interval based on 5,000 resamples.

Mediating effect of Presence of Meaning: As shown in Table 2, the age-adjusted total effect of fear of COVID-19 on PTSD was significant (*B* = 0.557, SE = 0.048, t = 11.504, *p* < 0.001, 95% CI [0.462, 0.652]). Fear of COVID-19 was negatively associated with Presence of Meaning (*B* = −0.157, SE = 0.033, t = −4.788, *p* < 0.001, 95% CI [−0.222, −0.093]). When Presence of Meaning was included in the outcome equation, it was negatively associated with PTSD (*B* = −0.373, SE = 0.040, t = −9.390, *p* < 0.001, 95% CI [−0.452, −0.295]), and the direct effect of fear remained significant (*B* = 0.499, SE = 0.047, t = 10.540, *p* < 0.001, 95% CI [0.406, 0.591]). The indirect effect through Presence of Meaning was significant (*B* = 0.059, BootSE = 0.015, 95% percentile bootstrap CI [0.031, 0.089]; Figure 1 and Table 4), indicating partial mediation after controlling for age.

**FIGURE 1:**
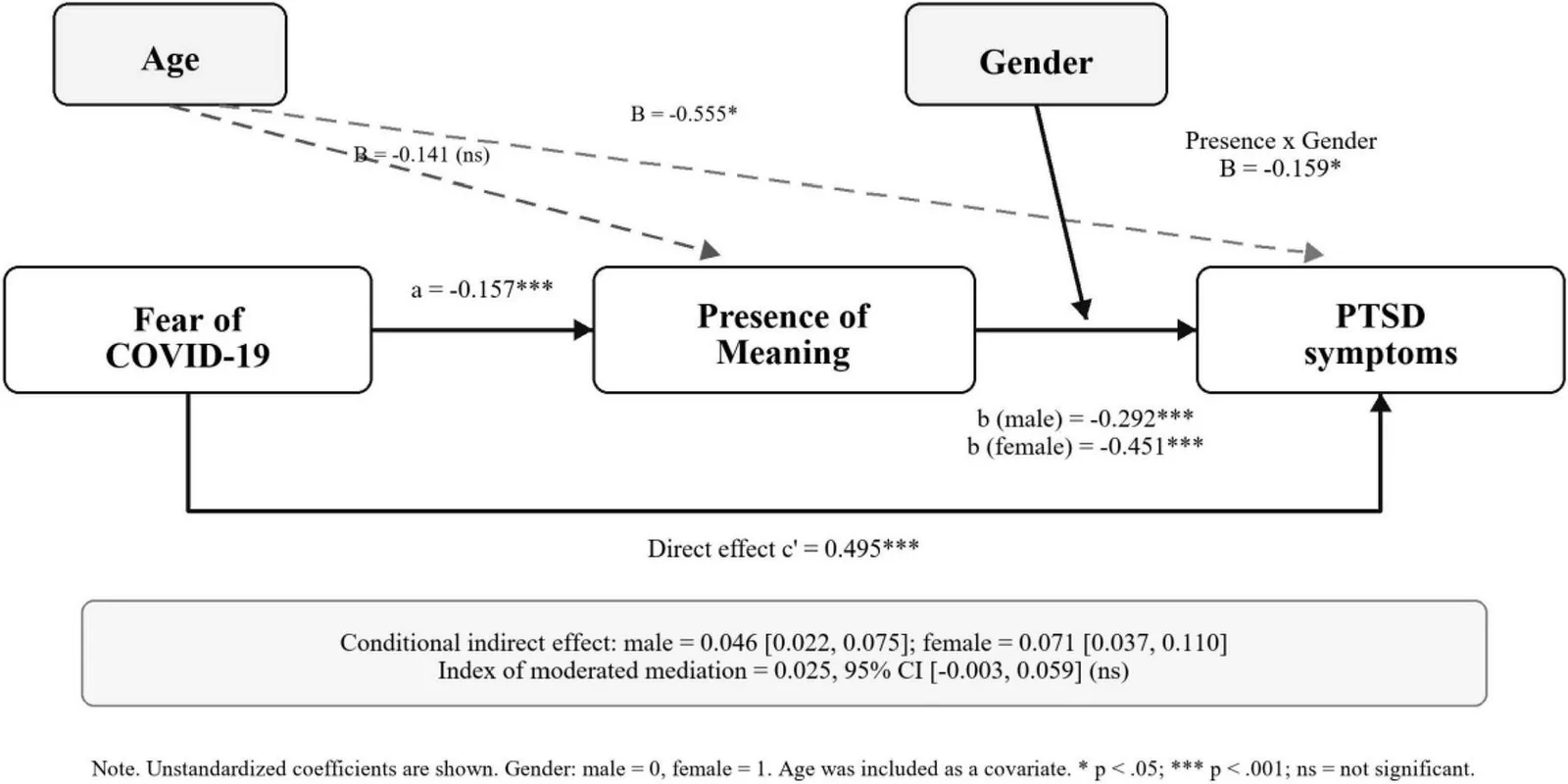
Graph of the moderating effect of gender in the presence of meaning mediating pathway.

Mediating effect of Search for Meaning: As shown in Table 3, fear of COVID-19 was not significantly associated with Search for Meaning after controlling for age (*B* = −0.052, SE = 0.028, t = −1.840, *p* = 0.066, 95% CI [−0.107, 0.003]). Search for Meaning was also not significantly associated with PTSD in the outcome equation (*B* = −0.093, SE = 0.048, t = −1.939, *p* = 0.053, 95% CI [−0.187, 0.001]), whereas the direct effect of fear remained significant (*B* = 0.553, SE = 0.048, t = 11.401, *p* < 0.001, 95% CI [0.457, 0.648]). The indirect effect through Search for Meaning was not significant (*B* = 0.005, BootSE = 0.004, 95% percentile bootstrap CI [−0.001, 0.015]; Figure 2 and Table 4). Thus, the age-adjusted simple mediation model did not support Search for Meaning as a mediator.

**FIGURE 2:**
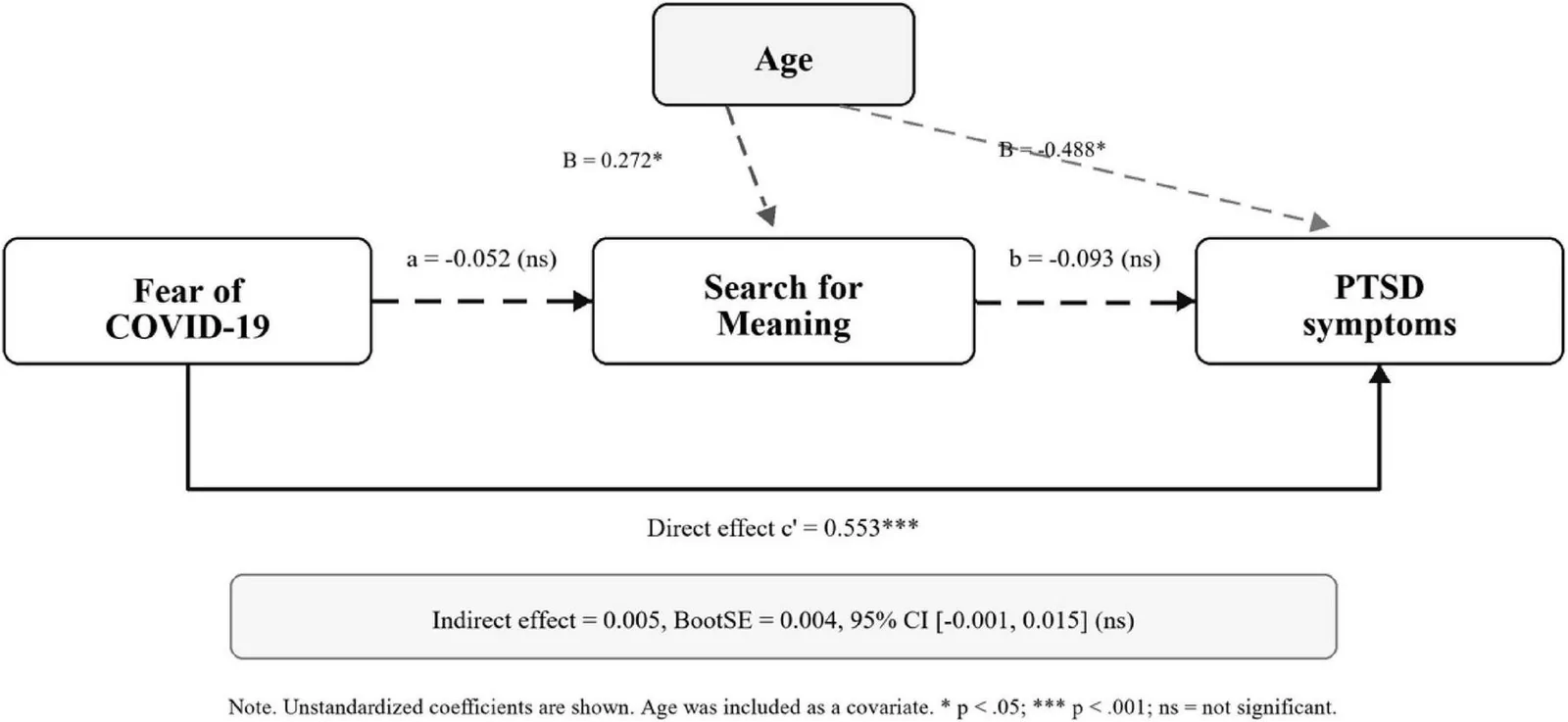
Simple mediation model of search for meaning in the fear–PTSD association.

### Parallel mediation model

Given the moderate correlation between Presence of Meaning and Search for Meaning (*r* = 0.29, *p* < 0.001), a parallel mediation model was conducted to estimate the unique indirect effect of each dimension while controlling for the other mediator and for age. The results are presented in Tables 5 and 6.

**TABLE 5:** Parallel mediation model.

Variable	Presence of meaning model			Search for meaning model			PTSD		
	*B*	*t*	*95% CI*	*B*	*t*	*95% CI*	*B*	*t*	*95% CI*
Fear of COVID-19	−0.157	−4.788***	[−0.222, −0.093]	−0.052	−1.840	[−0.107, 0.003]	0.499	10.547***	[0.406, 0.592]
Presence of meaning							−0.382	−9.200***	[−0.463, −0.300]
Search for meaning							0.034	0.709	[−0.061, 0.129]
Age	−0.141	−0.937	[−0.437, 0.154]	0.272	2.108*	[0.019, 0.525]	−0.577	−2.675**	[−1.000, −0.154]
*R* ^2^	0.018			0.006			0.157		
*F*	11.694***			4.137*			59.771***		

B = unstandardized regression coefficient; CI = 95% confidence interval. Age was included as a covariate in every equation.

* *p* < 0.05.

** *p* < 0.01.

*** *p* < 0.001.

**TABLE 6:** Indirect effects in parallel mediation model.

Effect	Effect (*B*)	BootSE	BootLLCI	BootULCI
Total indirect effect	0.058	0.015	0.032	0.090
Presence of meaning	0.060	0.015	0.032	0.092
Search for meaning	−0.002	0.003	−0.009	0.004

Effect (B) = unstandardized indirect effect; BootSE = bootstrap standard error; BootLLCI and BootULCI are the lower and upper limits of the 95% percentile bootstrap confidence interval based on 5,000 resamples.

As shown in Table 5, when both mediators and age were entered simultaneously, the direct effect of fear of COVID-19 on PTSD remained significant (*B* = 0.499, SE = 0.047, t = 10.547, *p* < 0.001, 95% CI [0.406, 0.592]). Presence of Meaning was negatively associated with PTSD (*B* = −0.382, SE = 0.042, t = −9.200, *p* < 0.001, 95% CI [−0.463, −0.300]), whereas Search for Meaning was not (*B* = 0.034, SE = 0.048, t = 0.709, *p* = 0.479, 95% CI [−0.061, 0.129]). The total indirect effect was significant (*B* = 0.058, BootSE = 0.015, 95% percentile bootstrap CI [0.032, 0.090]). The unique indirect effect through Presence of Meaning was significant (*B* = 0.060, BootSE = 0.015, 95% CI [0.032, 0.092]), whereas the unique indirect effect through Search for Meaning was not (*B* = −0.002, BootSE = 0.003, 95% CI [−0.009, 0.004]; Table 6). These results indicate that only Presence of Meaning served as a unique mediator in the age-adjusted parallel model.

### Testing for moderated mediation

To test H3a and H3b, two moderated mediation models (PROCESS Model 14) were estimated, one for each meaning dimension, with gender as a moderator of the second-stage mediator–PTSD path. Age was included as a covariate in all equations. Regression and conditional-effect results are presented in Tables 7, 8, 9 and 10.

**TABLE 7:** Age-adjusted model testing gender moderation of the presence of meaning–PTSD association.

Variable	Presence of meaning			PTSD		
	*B*	*t*	*95% CI*	*B*	*t*	*95% CI*
Fear of COVID-19	−0.157	−4.788***	[−0.222, −0.093]	0.495	10.458***	[0.402, 0.588]
Presence of Meaning				−0.292	−5.191***	[−0.402, −0.181]
Gender				0.391	0.672	[−0.751, 1.532]
Presence of Meaning × Gender				−0.159	−2.015*	[−0.314, −0.004]
Age	−0.141	−0.937	[−0.437, 0.154]	−0.555	−2.581*	[−0.977, −0.133]
*R* ^2^	0.018			0.159		
*F*	11.694***			48.729***		

B = unstandardized regression coefficient; CI = 95% confidence interval. Gender was dummy coded (male = 0, female = 1); × denotes the Presence of Meaning × Gender product term. Age was included as a covariate in both equations.

* *p* < 0.05.

** *p* < 0.01.

*** *p* < 0.001.

**TABLE 8:** Age-adjusted model testing gender moderation of the search for meaning–PTSD association.

Variable	Search for meaning			PTSD		
	*B*	*t*	*95% CI*	*B*	*t*	*95% CI*
Fear of COVID-19	−0.052	−1.840	[−0.107, 0.003]	0.553	11.388***	[0.457, 0.648]
Search for meaning				0.019	0.286	[−0.110, 0.147]
Gender				0.738	1.231	[−0.438, 1.913]
Search for Meaning × Gender				−0.231	−2.413*	[−0.420, −0.043]
Age	0.272	2.108	[0.019, 0.525]	−0.472	−2.127*	[−0.908, −0.037]
*R* ^2^	0.006			0.106		
*F*	4.137*			30.601***		

B = unstandardized regression coefficient; CI = 95% confidence interval. Gender was dummy coded (male = 0, female = 1); × denotes the Search for Meaning × Gender product term. Age was included as a covariate in both equations.

*p < 0.05.

**p < 0.01.

***p < 0.001.

**TABLE 9:** Conditional b-path and conditional indirect effects for the presence of meaning pathway.

Gender	b-path	SE	t	95% CI	Conditional indirect effect	BootSE	BootLLCI	BootULCI
Male	−0.292	0.056	−5.191***	[−0.402, −0.181]	0.046	0.013	0.022	0.075
Female	−0.451	0.056	−8.038***	[−0.561, −0.341]	0.071	0.019	0.037	0.110

Index of moderated mediation = 0.025, BootSE = 0.015, 95% percentile bootstrap CI [−0.003, 0.059]. The b-path confidence intervals are conventional 95% confidence intervals. Conditional indirect effects and the index of moderated mediation use 95% percentile bootstrap confidence intervals based on 5,000 resamples. Age was included as a covariate.

*p < 0.05.

**p < 0.01.

***p < 0.001.

**TABLE 10:** Conditional b-path and conditional indirect effects for the search for meaning pathway.

Gender	b-path	SE	*t*	95% CI	Conditional indirect effect	BootSE	BootLLCI	BootULCI
Male	0.019	0.066	0.286	[−0.110, 0.147]	−0.001	0.004	−0.009	0.006
Female	−0.213	0.070	−3.034**	[−0.350, −0.075]	0.011	0.008	−0.001	0.030

Index of moderated mediation = 0.012, BootSE = 0.009, 95% percentile bootstrap CI [−0.002, 0.034]. The b-path confidence intervals are conventional 95% confidence intervals. Conditional indirect effects and the index of moderated mediation use 95% percentile bootstrap confidence intervals based on 5,000 resamples. Age was included as a covariate.

*p < 0.05.

**p < 0.01.

***p < 0.001.

As shown in Table 7, the interaction between Presence of Meaning and gender was significant in the PTSD equation (*B* = −0.159, SE = 0.079, t = −2.015, *p* = 0.044, 95% CI [−0.314, −0.004]), whereas the gender main effect was not (*B* = 0.391, SE = 0.582, t = 0.672, *p* = 0.502, 95% CI [−0.751, 1.532]). Simple-slope analyses (Table 9) showed a significant negative association for both males (*B* = −0.292, SE = 0.056, t = −5.191, *p* < 0.001, 95% CI [−0.402, -0.181]) and females (*B* = −0.451, SE = 0.056, t = −8.038, *p* < 0.001, 95% CI [−0.561, -0.341]), with a stronger negative slope among females (Figure 3).The conditional indirect effects through Presence of Meaning were significant for males (*B* = 0.046, BootSE = 0.013, 95% CI [0.022, 0.075]) and females (*B* = 0.071, BootSE = 0.019, 95% CI [0.037, 0.110]; Table 9). However, the index of moderated mediation was not significant (index = 0.025, BootSE = 0.015, 95% CI [−0.003, 0.059]).

**FIGURE 3:**
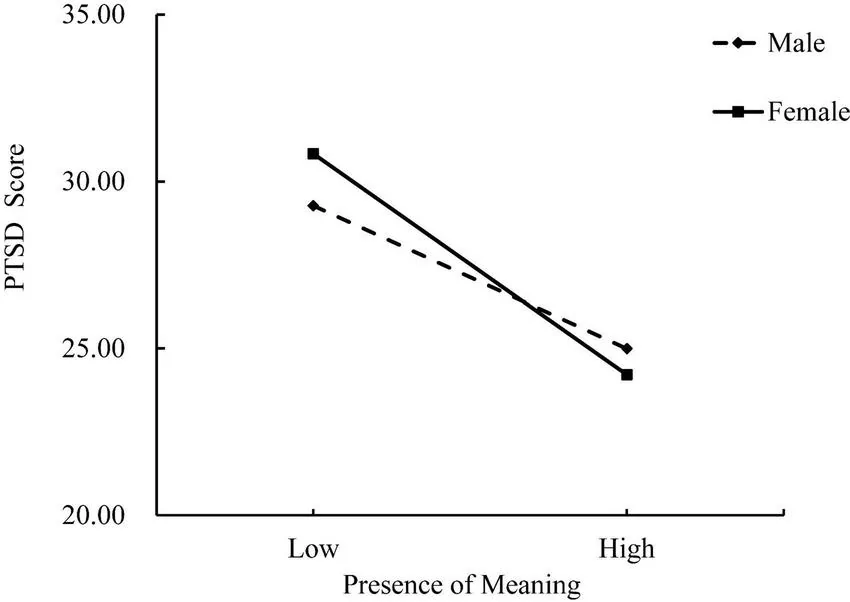
Interaction between presence of meaning and gender in predicting PTSD symptoms after adjustment for age.

As shown in Table 8, the interaction between Search for Meaning and gender was significant in the PTSD equation (*B* = −0.231, SE = 0.096, t = −2.413, *p* = 0.016, 95% CI [−0.420, -.043]), whereas the gender main effect was not (*B* = 0.738, SE = 0.599, t = 1.231, *p* = 0.218, 95% CI [−0.438, 1.913]). Simple-slope analyses (Table 10) showed that the Search–PTSD association was not significant among males (*B* = 0.019, SE = 0.066, t = 0.286, *p* = 0.775, 95% CI [−0.110, 0.147]) but was significantly negative among females (*B* = −0.213, SE = 0.070, t = −3.034, *p* = 0.002, 95% CI [−0.350, -0.075]). However, the conditional indirect effects were not significant for either males (*B* = −0.001, BootSE = 0.004, 95% CI [−0.009, 0.006]) or females (*B* = 0.011, BootSE = 0.008, 95% CI [−0.001, 0.030]), and the index of moderated mediation was not significant (index = 0.012, BootSE = 0.009, 95% CI [−0.002, 0.034]).

Thus, H1 and H2a were supported, whereas H2b was not. H3a received qualified support: the second-stage interaction was significant, but the moderated mediation index was not. H3b also received qualified support: the Search × Gender interaction was significant, but the conditional indirect effects and the index of moderated mediation were not significant.

## Discussion

The present study examined age-adjusted associations among fear of COVID-19, PTSD symptoms, and two dimensions of meaning in life in adolescents. Simple and parallel mediation models distinguished Presence of Meaning from Search for Meaning, and two Model 14 analyses tested whether gender moderated the second-stage paths from Presence of Meaning and Search for Meaning to PTSD.

### Fear of COVID-19 and PTSD in Chinese adolescents during pandemic lockdowns

Consistent with H1, fear of COVID-19 was positively correlated with PTSD symptoms (*r* = 0.31, *p* < 0.001), and its age-adjusted total effect was significant (*B* = 0.557, *p* < 0.001). The model containing fear of COVID-19 and age accounted for 9.9% of the variance in PTSD symptoms (R^2^ = 0.099). This pattern is consistent with pandemic studies reporting associations between fear or perceived threat and PTSD symptoms (Crosta et al., 2020; González-Sanguino et al., 2020). Because the model was cross-sectional and included age, the explained variance cannot be attributed to fear alone and the coefficient does not establish temporal precedence or causation.

The observed association is compatible with emotional processing theory, which emphasizes the role of pathological fear structures in PTSD (Foa and Kozak, 1986). Repeated pandemic-related threat cues could be relevant to such fear structures, but the present study did not measure fear-network formation, media exposure, or changes over time. The data therefore show that greater fear and more severe PTSD symptoms co-occurred; they do not demonstrate that fear consolidated into a pathological network or caused the symptoms. Moreover, because data collection spanned November and December 2022, participants may have encountered different policy and infection contexts during the collection window.

Developmental considerations may also be relevant. Adolescence involves heightened emotional reactivity, sensitivity to social evaluation, and continuing maturation of regulatory systems (Casey et al., 2010; Steinberg, 2008). Disruption of social and academic routines may have made fear regulation more difficult for some adolescents (Sayed et al., 2021; Sultana et al., 2021), while the salience of peer relationships could have increased the burden of reduced in-person contact (Steinberg and Morris, 2001). These possibilities were not directly measured and should be treated as hypotheses for future longitudinal research.

It is also worth noting that the mean PTSD symptom score in our sample was 27.38 (SD = 11.30), and 72 participants (5.6%) met the clinical screening cutoff of ≥ 50 on the PCL-C, indicating probable PTSD. This prevalence rate is comparable to previous studies reporting PTSD symptoms among adolescents during the COVID-19 pandemic (Cao et al., 2022), and underscores the importance of examining PTSD symptoms dimensionally in general adolescent populations, rather than focusing solely on diagnostic thresholds. The fact that over 5% of a community-based adolescent sample screened positive for probable PTSD highlights the substantial mental health burden of the pandemic on young populations and the need for accessible mental health support in school settings.

### The mediating role of meaning in life: presence versus search

A central contribution of this study is the direct comparison of Presence of Meaning and Search for Meaning. In both simple and parallel age-adjusted models, Presence of Meaning showed a significant indirect effect, whereas Search for Meaning did not. This pattern demonstrates that the two MLQ dimensions should not be treated as interchangeable, but it does not establish a causal mediating mechanism in cross-sectional data.

Presence of Meaning, defined as an existing sense that life has purpose, coherence, and significance (Steger et al., 2006), showed significant indirect effects in the simple (*B* = 0.059, 95% CI [0.031, 0.089]) and parallel (*B* = 0.060, 95% CI [0.032, 0.092]) age-adjusted models. Fear of COVID-19 was negatively associated with Presence of Meaning (*B* = −0.157, *p* < 0.001), which was in turn negatively associated with PTSD symptoms in the simple (*B* = −0.373, *p* < 0.001) and parallel (*B* = −0.382, *p* < 0.001) models. This pattern is compatible with meaning-making theory (Park, 2010). Daily meaning salience has also been associated with physical activity in previously inactive exercise initiates (Hooker and Masters, 2018), suggesting that awareness of meaning may be linked to broader patterns of adaptive functioning, although this does not establish the specific mechanism observed here. Accordingly, Presence of Meaning was the only meaning-related dimension with a significant unique indirect association in this sample.

In contrast, Search for Meaning, defined as the active effort to find or discover meaning and purpose in life (Steger et al., 2006), did not significantly mediate the fear-PTSD association. The simple indirect effect was not significant (*B* = 0.005, 95% CI [−0.001, 0.015]), and the unique indirect effect in the parallel model was also not significant (*B* = −0.002, 95% CI [−0.009, 0.004]). Consistent with these indirect-effect tests, fear of COVID-19 was not significantly associated with Search for Meaning in the age-adjusted mediator equation (*B* = −0.052, *p* = 0.066); Search for Meaning was not significant in either the simple PTSD equation (*B* = −0.093, *p* = 0.053) or the parallel PTSD equation (*B* = 0.034, *p* = 0.479). The shift in the Search coefficient after Presence of Meaning was included is consistent with shared variance between the two dimensions (*r* = 0.29), but the data provide no evidence that Search for Meaning independently transmitted the association between fear and PTSD.

Search for Meaning did not show a significant simple or unique parallel indirect effect. One possible interpretation is that active searching is a process rather than an already available resource, but the cross-sectional data do not test this mechanism (Steger et al., 2009). We also did not estimate a Presence of Meaning × Search for Meaning interaction. Therefore, the results do not support conclusions about a “search–presence gap,” do not show that searching is ineffective when Presence is low, and should not be generalized beyond the models that were actually tested.

Another complementary explanation draws from the distinction between “meaning-making” as a process versus “meaning-made” as an outcome (Park, 2010). During an acute crisis such as the pandemic, individuals may have limited cognitive and emotional resources to engage in the effortful process of searching for meaning. Instead, pre-existing meaning structures, what Park (2010) termed “global meaning”, may be more immediately accessible and effective in buffering stress responses. Adolescents with a strong pre-existing sense of meaning likely had a well-developed cognitive framework for interpreting and integrating stressful experiences, whereas those without such a framework may have been too overwhelmed by acute stress to initiate an effective search process. This interpretation is consistent with research on coping flexibility, which suggests that under high-stress conditions, individuals tend to rely on established coping resources rather than novel or effortful strategies (Bonanno and Burton, 2013).

Furthermore, the developmental context of adolescence is relevant here. Adolescence is a critical period for identity exploration and meaning construction (Erikson, 1968; Bronk, 2011). For many adolescents, the active search for meaning is a normative developmental process that may not yet have yielded a stable sense of meaning. In our sample, Search scores (M = 26.13, SD = 6.25) were similar to Presence scores (M = 23.54, SD = 7.34), and the correlation between them was moderate (*r* = 0.29), suggesting that many adolescents were actively engaged in meaning-seeking while also possessing some sense of meaning. However, under the acute stress of the pandemic, the active search process may have been disrupted without providing immediate protective benefits. These findings raise the possibility, as a hypothesis for future research, that supporting an existing sense of meaning may be worth investigating alongside meaning-seeking at developmentally appropriate stages. Whether such support would reduce PTSD symptoms remains to be tested in longitudinal or intervention research.

### The moderating role of gender

Descriptive comparisons showed that gender differences in the primary study variables were relatively small, with females reporting slightly higher PTSD symptoms and fear of COVID-19, and males reporting slightly higher Presence of Meaning and Search for Meaning (see Table 1). The gender main effect on PTSD was not significant (B = 0.391, *p* = 0.502), suggesting that the observed moderation of the Presence–PTSD path is not simply an artifact of mean-level gender differences. Gender moderated the age-adjusted Presence of Meaning-PTSD association (interaction *B* = −0.159, *p* = 0.044): the negative slope was stronger among females (*B* = −0.451) than males (*B* = −0.292). One possible explanation is that adolescent girls’ meaning systems may be more relationally embedded (Rose and Rudolph, 2006), making the maintenance of meaning during social disruption particularly protective. However, this interpretation is speculative, as we did not directly measure relational identity or sources of meaning.

Notably, the index of moderated mediation was not significant (index = 0.025, 95% CI [−0.003, 0.059]), indicating that the overall indirect effect (fear → Presence → PTSD) did not significantly differ by gender. This is likely because the gender difference was specific to the b-path (Presence → PTSD), rather than the a-path (Fear → Presence) or the indirect effect as a whole. Thus, while the meaning-to-symptom path was stronger for females, the full mediating process operated similarly across genders.

This pattern is partially consistent with prior research showing that meaning in life is a robust protective factor for mental health (Arslan et al., 2022; Fischer et al., 2023). However, few studies have directly tested gender differences in this association among adolescents. The present findings suggest that gender may be a relevant boundary condition, but the non-significant moderated mediation index cautions against overinterpreting this difference as evidence of gender-specific mechanisms.

Search for Meaning showed no significant indirect effect, highlighting the distinction between having meaning and seeking meaning. This pattern suggests that the two dimensions may function differently during acute stress, but it does not establish that strengthening Presence of Meaning would reduce PTSD symptoms. Because the moderated mediation index was not significant, any claim that females would benefit more from meaning-focused interventions would go beyond the current evidence.

We also examined whether gender moderated the Search for Meaning pathway (H3b). Although the unconditional indirect effect through Search for Meaning was not significant, the Search × Gender interaction was significant (*B* = −0.231, *p* = 0.016). Simple-slope analyses showed that the Search–PTSD association was not significant among males (*B* = 0.019, *p* = 0.775) but was significantly negative among females (*B* = −0.213, *p* = 0.002). This pattern suggests that active meaning-seeking may be differentially associated with PTSD symptoms for adolescent girls and boys. However, because the fear-to-Search path was not significant, the conditional indirect effects were not significant for either gender, and the index of moderated mediation was not significant (index = 0.012, 95% CI [−0.002, 0.034]). Thus, the gender difference was confined to the b-path rather than the overall mediated process. This pattern parallels the Presence of Meaning findings and reinforces the distinction between path-level moderation and moderated mediation. Theoretically, girls who are actively searching for meaning during acute stress may be more vulnerable to distress if that search does not yield a stable sense of meaning (Park, 2010; Steger et al., 2009). Alternatively, the negative association among females could reflect a more ruminative or less effective search process. These interpretations remain speculative, and the cross-sectional design cannot determine whether gender differences in the Search–PTSD path are causally related to PTSD symptoms. Future longitudinal research is needed to clarify these patterns.

In sum, gender moderated the second-stage paths from both Presence of Meaning and Search for Meaning to PTSD, but the overall indirect effects did not differ by gender. These findings underscore the importance of distinguishing between path-level moderation and overall moderated mediation, and highlight the need for future research to directly measure the psychological processes underlying gender differences in meaning-making and trauma responses.

### Limitations

Despite its contributions, several limitations of this study warrant consideration. First, the cross-sectional design prevents definitive conclusions regarding causality and the temporal dynamics of the relationships between fear, meaning in life, and PTSD. While our hypothesized model is theoretically grounded in emotional processing theory (Foa and Kozak, 1986), terror management theory (Pyszczynski et al., 2015), and meaning-making theory (Park, 2010), alternative directional models are equally plausible. For instance, individuals with pre-existing subclinical PTSD symptoms may have been more likely to report heightened fear of COVID-19 (Fischer et al., 2023; Blechert et al., 2007), and the relationship between meaning and PTSD could be bidirectional (Park, 2010). Future longitudinal studies with multiple time points, particularly cross-lagged panel designs or latent growth curve modeling, are essential to disentangle these directional pathways.

Second, several measurement and analysis limitations should be noted. The PCL-C assesses responses to stressful experiences without anchoring them to a specific DSM-5 Criterion A event; although participants were instructed to reference COVID-19-related experiences, the symptoms may not have been exclusively pandemic-related. The screening threshold did not provide a clinical diagnosis. Future work should use event-anchored instruments or clinical interviews. Reliance on self-report measures may introduce common method bias, and the Harman single-factor test has limited sensitivity (Podsakoff et al., 2003). The MLQ configural model had acceptable but not optimal fit (CFI = 0.936, RMSEA = 0.103), although metric and scalar invariance were fully supported. Finally, stochastic regression single imputation produced one completed dataset and may understate uncertainty because imputation variability is not propagated. Future studies should use designs and missing-data methods that better quantify these sources of uncertainty.

Third, convenience sampling from four schools limits generalizability. Excluding participants with significant pre-pandemic trauma narrowed the target sample, and a systematic retained-versus-excluded comparison was not available because demographic information (gender and age) among excluded cases was often incomplete in an unsystematic manner, preventing a reliable statistical comparison. Selection bias therefore cannot be completely ruled out. This represents a significant data management limitation that we acknowledge with regret. The associations may differ across developmental stages, clinical populations, regions, and adolescents with multiple trauma histories. Replication in more diverse probability-based and clinically characterized samples is needed.

Fourth, while we adopted a minimal-disclosure approach for participants who screened positive for probable PTSD (i.e., providing general information about available school-based psychological support through routine school communication channels, without disclosing specific screening scores or clinical details), we did not explicitly disclose this potential limit to confidentiality during the informed consent process. We also did not conduct systematic follow-up assessments to evaluate whether families made use of the provided support resources. These omissions represent both an ethical and a procedural limitation, which we acknowledge with regret. Future research should clearly state in the informed consent form that researchers may provide general information about available support resources through routine school channels when participants’ responses indicate potential psychological risk, and should incorporate structured follow-up protocols to ensure that at-risk adolescents receive appropriate care.

Fifth, gender was measured using only the binary response options male and female, which does not capture the spectrum of gender identity or relevant social experiences (Kimerling et al., 2018). In the Model 14 analyses, gender entered only the PTSD equation, so the fear-to-mediator a paths were pooled across groups. Additionally, the data collection period (November–December 2022) coincided with a major policy shift in China’s COVID-19 response; because individual completion dates were not recorded, we could not test for period effects, and unmodeled temporal heterogeneity may limit interpretation. Finally, the Presence × Search interaction was not tested. Future studies should use more inclusive gender measures, record exact dates, model period effects, and test interactions between meaning dimensions when theoretically justified.

## Conclusion

After adjusting for age, fear of COVID-19 was positively associated with PTSD symptoms, and Presence of Meaning, but not Search for Meaning, showed a significant indirect association. Gender moderated the second-stage paths from both Presence of Meaning and Search for Meaning to PTSD, but the overall indirect effects did not differ by gender. These cross-sectional findings underscore the distinction between having meaning and seeking meaning. They cannot establish whether these associations are causal or whether interventions targeting meaning would reduce PTSD symptoms; longitudinal and experimental research is needed to address those questions.

## Data Availability

The original contributions presented in the study are included in the article/Supplementary material, further inquiries can be directed to the corresponding author.
