# Supplementary material for: Fear of COVID-19 and PTSD symptoms in adolescents: the mediating role of meaning in life and the moderating role of gender
Source: Front Psychol. 2026 Oct 23;17:1912062. doi: 10.3389/fpsyg.2026.1912062 (PMC13739693; doi:10.3389/fpsyg.2026.1912062)
Supplement: Supplementary file 1 [file Data_sheet_1.zip › S1-S7/S1-Data log.docx]

**Data Log: From Raw Data to Analyzed Dataset**

**Research Topic:** Fear of COVID-19 and PTSD Symptoms in Adolescents: The Mediating Role of Meaning in Life and the Moderating Role of Gender

**Data Collection Period:** November-December 2022

**Data Collection Method:** Paper-based questionnaires (four schools in Guizhou, Henan, and Hubei)

**Data Entry Tool:** EpiData

**Data Format:** Raw Data: Excel (.xlsx) → Analysis Data: SPSS (.sav)

**1 Preliminary Preparations for Data Cleaning**

**1.1 Data Entry Verification**

1.1.1 Procedures

Data was entered using EpiData, exported to Excel format, and imported into SPSS 27.0. Each entered value was cross-checked against the original paper questionnaires, with a focus on identifying the following issues:

(1) Incorrect entry locations (e.g., entering the answer to Question 5 into Question 6)

(2) Inconsistent item coding (e.g., scales that should be coded 1-5 but contain values outside this range, such as 6 or 7)

(3) Standardize variable coding rules:

- Categorical variables (e.g., gender): Assign clear values, where 1 = male and 2 = female;
- Scale items: 1 = “Strongly Disagree/Never” to 5/7 = “Strongly Agree/Always”;
- Uniformly mark missing values: All blank, uncompleted items are marked as system missing values (SYSMIS)

1.1.2 Verification Results

(1) A small number of data entry errors (<5) were identified and corrected one by one based on the original questionnaires

(2) All variables have been uniformly coded; values outside the range have been corrected or converted to missing values

(3) Sample size: N = 1,400 (no exclusions)

**2 Exclusion of Invalid Questionnaire Samples**

**2.1 Exclusion Criterion 1: History of Severe Mental Disorders**

2.1.1 Procedure: Screening was conducted based on the questionnaire item “Have you ever been diagnosed by a professional medical institution with a severe mental disorder (e.g., schizophrenia, bipolar disorder, etc.)?”

2.1.2 Rationale: A history of severe mental disorders may independently influence PTSD symptoms and interfere with this study’s assessment of COVID-19-specific effects. For the purposes of this study, participants with such a history do not meet the inclusion criteria and should be excluded.

2.1.3 Number Excluded: 0

2.1.4 Remaining Sample: N = 1,400

**2.2 Exclusion Criterion 2: History of Major Trauma**

2.2.1 Procedure: Screen based on the questionnaire item “Have you experienced a major traumatic event (e.g., natural disaster, major accident, abuse, etc.) prior to the COVID-19 pandemic?”

2.2.2 Rationale: To ensure clear attribution in the assessment of PTSD symptoms, this study focuses on COVID-19-related psychological reactions. Participants with a history of major trauma may exhibit PTSD symptoms stemming from pre-pandemic events rather than the pandemic itself; therefore, they are excluded to enhance internal validity.

2.2.3 Number of participants excluded: 19

2.2.4 Remaining sample: N = 1,381

**2.3 Exclusion Criterion 3: Extensive Missing Data**

2.3.1 Procedure: Identify questionnaires with a missing item rate exceeding 10% on the main scales (FCV-19S, PCL-C, MLQ).

2.3.2 Rationale: According to data cleaning standards, when more than 10% of the key variables in a single questionnaire are missing, statistical imputation cannot effectively restore data quality. The entire questionnaire lacks analytical value and should be excluded rather than “rescued” through imputation.

2.3.3 Number of Participants Excluded: 49

2.3.4 Remaining Sample: N = 1,332

**2.4 Exclusion Criterion 4: Incomplete Questionnaires**

2.4.1 Procedure: Exclude invalid questionnaires with extensive omissions or obvious patterns in responses (e.g., missing key demographic variables, selecting the same option for all items, or alternating in a cyclical pattern such as “131313”). (In this study, “patterned responses” are defined as selecting the same score for every item in the entire questionnaire or exhibiting a mechanical alternating pattern (e.g., 1-3-1-3). Such response patterns indicate that the participant did not read the questions carefully, and the data lack genuine response variation; therefore, the entire questionnaire should be discarded.)

2.4.2 Rationale: Severely incomplete questionnaires or responses exhibiting obvious patterns indicate that participants did not cooperate in good faith; the data quality is unreliable and cannot be corrected through statistical methods; such questionnaires should be discarded outright.

2.4.3 Number of participants excluded: 41

2.4.4 Remaining sample: N = 1,291 (final analysis sample)

**3 Standardized Handling of Missing Values**

**3.1 Diagnosis of Missing Values**

3.1.1 Procedure: For the 1,291 questionnaires ultimately retained, calculate the missing value rates for each scale (FCV-19S, PCL-C, MLQ-PM, MLQ-SM) and each demographic variable.

3.1.2 Diagnostic Results: The missing rate for items on the main scales was <2%

**3.2 Selection of Imputation Method**

In accordance with standard data cleaning guidelines, stochastic regression imputation was used in this analysis. The “Analyze → Multiple Imputation → Imputate Missing Values” function in SPSS 27.0 was utilized, with the specific procedure as follows:

(1) All items from each scale (7 items from the FCV-19S, 17 items from the PCL-C, and 10 items from the MLQ) were included in the list of predictor variables for the imputation model, and regression imputation was performed using the covariance structure among the items

(2) Add a random residual term (with residuals following a normal distribution with a mean of 0) to the predicted values to preserve the natural variability of the data.

(3) Set the number of imputations to 5 to generate 5 imputed datasets.

(4) Calculate Cronbach’s α for each scale separately across the 5 datasets to assess whether the randomness of the imputation affects the stability of the scale structure.

**3.3 Verification of Interpolation Stability**

| Scale | Cronbach’s α Range Across 5 Datasets | Stability Conclusion |
| --- | --- | --- |
| FCV-19S | 0.89 - 0.91 | Minimal fluctuation |
| PCL-C | 0.92 - 0.94 | Minimal fluctuation |
| MLQ-PM | 0.89 - 0.91 | Minimal fluctuation |
| MLQ-SM | 0.85 - 0.87 | Minimal fluctuation |

Given the high consistency in reliability across the datasets, the randomness of the interpolation had no substantial impact on the data structure. The third imputed dataset, whose Cronbach’s α values fell at the median level among all imputed datasets and which was therefore considered representative, was selected as the final dataset for analysis.

**4 Variable Calculations**

**4.1 Total PTSD Symptom Score (PCL-C)**

(1) Calculation method: Sum of scores for 17 items (each item scored 1-5 points)

- Correction Note: The SPSS calculation syntax for the PCL-C total score in the second draft contained an error (the formula `COMPUTE PCL_C = SUM(...)` was incorrect). Due to the author’s oversight, only the first 10 items of the scale were calculated, resulting in a systematic underestimation of the total score (M = 15.36, SD = 6.48). The descriptive statistics for the erroneous version in the second draft are as follows (erroneous version):

Table 1 Descriptive Statistics for the PCL-C Total Score (Erroneous Version)

|  | *N* | *Min* | *Max* | *M* | *SD* |
| --- | --- | --- | --- | --- | --- |
| PTSD | 1291 | 10 | 50 | 15.36 | 6.48 |

This error was identified during the audit of the third draft, and the scores have been recalculated using the correct formula (the `SUM.17` function was used to ensure all 17 items were included).

- Corrected results: PCL-C total score range 17-85 (theoretical range 17-85), M = 27.38, SD = 11.30.
- Clinical cutoff score: ≥50 indicates a possible PTSD positive result; 72 participants (5.6%) met this criterion.

Table 2 Descriptive Statistics for PCL-C Total Scores (Correct Version)

|  | *N* | *Min* | *Max* | *M* | *SD* |
| --- | --- | --- | --- | --- | --- |
| PTSD | 1291 | 17 | 81 | 27.38 | 11.30 |

**4.2 Total COVID-19 Fear Score (FCV-19S)**

(1) Calculation method: Sum of scores for 7 items (1-5 points per item); total score range 7-35; M = 15.25; SD = 6.18.

**4.3 Meaning in Life Questionnaire (MLQ) Score**

- Correction Note: Upon re-verifying the item-dimension correspondences in the Chinese-revised version of the MLQ by Chen et al. (2015), it was discovered that items 8 and 10 of the MLQ were incorrectly swapped in the initial analysis (in the second draft, MLQ-8 was assigned to “Presence of Meaning,” and MLQ-10 to “Search for Meaning”). According to the original literature, the correct assignment is as follows: “Presence of Meaning”: Items 2, 4, 7, 9, and 10 (with Item 2 reverse-scored); “Search for Meaning”: Items 1, 3, 5, 6, and 8. The scores were recalculated after the correction.
- Reverse Scoring: Transformation—recoded into different variables—new values versus old values (1-7, 2-6, 3-5, 4-4, 5-3, 6-2, 7-1)
- Presence of Meaning: Transformation—calculated variable—total score, M = 23.54, SD = 7.34
- Search for Meaning: Transformation—Calculated Variables—Total Score, M = 26.13, SD = 6.25

**5 Review of Validity at the Total Score Level**

(1) After completing the item-level outlier check (see 1.1), sample screening, missing value imputation, and scale total score calculation, a final comparison of the theoretical and actual ranges of each scale’s total score is conducted as the final validation of data quality.

(2) Verification Conclusion: All scale total scores fall within the theoretical range, and no deviations in total scores due to outliers were found. Combined with the record in Section 1.1 indicating no out-of-range values at the item level, the data quality is confirmed to be reliable.

**6 The data has been confirmed to be clean, and a final analysis data file has been generated for subsequent statistical analysis.**

**7 Confirmation of Core Compliance Standards**

- All data cleaning operations are well-documented and fully recorded
- No qualified samples were arbitrarily deleted for any reason (including the pursuit of statistical significance)
- No original response values were altered
- Interpolation was based on statistical standards and was not arbitrarily fabricated
- All 109 excluded participants had clear, traceable reasons for exclusion
- Subsequent statistical analysis may proceed only after the dataset has been verified as error-free
