# Supplementary material for: Fear of COVID-19 and PTSD symptoms in adolescents: the mediating role of meaning in life and the moderating role of gender
Source: Front Psychol. 2026 Oct 23;17:1912062. doi: 10.3389/fpsyg.2026.1912062 (PMC13739693; doi:10.3389/fpsyg.2026.1912062)
Supplement: Supplementary file 1 [file Data_sheet_1.zip › S1-S7/S5-Scoring verification and analysis outputs.docx]

**S5 Scoring verification and analysis outputs**

S5.1 Verification of recalculated total scores

| Variable | *N* | *Min* | *Max* | *Mean* | *SD* |
| --- | --- | --- | --- | --- | --- |
| diff-PTSD | 1291 | 0 | 0 | 0 | 0 |
| diff-Fear | 1291 | 0 | 0 | 0 | 0 |
| diff-Presence | 1291 | 0 | 0 | 0 | 0 |
| diff-Search | 1291 | 0 | 0 | 0 | 0 |

Note: The difference is the total score in S2 minus the total score recalculated based on project-level variables. All differences are 0, and the correlation between the corresponding total score and the recalculated total score is 1.000, indicating that the scoring procedure is correct.

S5.2 Descriptive statistics

| Variable | *N* | *Min* | *Max* | *Mean* | *SD* |
| --- | --- | --- | --- | --- | --- |
| Age | 1291 | 11 | 18 | 14.13 | 1.35 |
| PTSD | 1291 | 17 | 81 | 27.38 | 11.30 |
| Fear | 1291 | 7 | 35 | 15.25 | 6.18 |
| Presence | 1291 | 5 | 35 | 23.54 | 7.34 |
| Search | 1291 | 5 | 35 | 26.13 | 6.25 |

S5.3 Reliability

| Scale | Items | Cronbach’s α |
| --- | --- | --- |
| PCL-C | 17 | 0.93 |
| FCV-19S | 7 | 0.90 |
| MLQ-Presence | 5 | 0.90 |
| MLQ-Search | 5 | 0.86 |
| MLQ | 10 | 0.86 |

S5.4 Correlations

| Variable | 1 | 2 | 3 | 4 |
| --- | --- | --- | --- | --- |
| 1.PTSD | 1 |  |  |  |
| 2. Fear | 0.31^**^ | 1 |  |  |
| 3. Presence | -0.28^*^^*^ | -0.13^**^ | 1 |  |
| 4. Search | -0.07^*^ | -0.05 | 0.29 | 1 |

Note: **p < .05, **p < .01.*
