# Supplementary material for: Fear of COVID-19 and PTSD symptoms in adolescents: the mediating role of meaning in life and the moderating role of gender
Source: Front Psychol. 2026 Oct 23;17:1912062. doi: 10.3389/fpsyg.2026.1912062 (PMC13739693; doi:10.3389/fpsyg.2026.1912062)
Supplement: Supplementary file 1 [file Data_sheet_1.zip › S1-S7/S7-Supplementary Materials Index.docx]

**S7. Supplementary Materials Index**

| Material | File name | Content | Location in manuscript | Related comment |
| --- | --- | --- | --- | --- |
| S1 | S1-Data log.docx | Complete data-cleaning log from raw data (N = 1,400) to final analytic dataset (N = 1,291), including exclusion criteria, missing-data diagnosis, multiple imputation, and correction records | Methods | Comment 1 |
| S2 | S2-Data. sav / .csv | De-identified final analytic dataset containing item-level variables, computed total scores, and analysis variables | Methods | Comment 1 |
| S3 | S3-Scoring and Data Processing Syntax.sps | SPSS syntax verifying reverse scoring, MLQ item assignment, PCL-C 17-item total, FCV-19S total, reliability, and correlations from S2 onward | Methods; Supplementary S5 | Comment 1 |
| S4 | S4-Variable Codebook.xlsx | Variable names, labels, coding rules, item-to-dimension mappings, and scoring formulas | Methods: Measures | Comment 1 |
| S5 | S5-Scoring verification and analysis outputs.docx | Outputs verifying that recalculated total scores are identical to those in S2 (all differences = 0; correlations = 1.000), plus descriptives, reliability, and correlations | Results; Methods | Comment 1 |
| S6 | S6-MLQ measurement invariance lavaan.R /txt | Supplementary Material S7. Mplus syntax (.inp) and outputs (.out) for the measurement-invariance models, provided as a cross-validation of the lavaan results reported in the main text | Methods: Measurement invariance; Results | Comment 2 |
| S7 | S7-Supplementary Materials Index.docx | This index of all supplementary materials | All | All comments |
